# Cognitive Functioning in Toxic Oil Syndrome Survivors: A Case-Control Study Four Decades After the Epidemic

**DOI:** 10.3390/jcm14113746

**Published:** 2025-05-27

**Authors:** José Lapeña-Motilva, Mariano Ruiz-Ortiz, Glen M. Doniger, María Antonia Nogales, Verónica Giménez de Bejar, Sonia Álvarez-Sesmero, Montserrat Morales, Fernando Bartolomé, Carolina Alquézar, Durjoy Lahiri, Cecilia García-Cena, Julián Benito-León

**Affiliations:** 1 Department of Neurology, 12 de Octubre University Hospital, 28041 Madrid, Spain; joselapemo@gmail.com (J.L.-M.); mariano.ruiz.ortiz@gmail.com (M.R.-O.); neuro.gimenezdebejar@gmail.com (V.G.d.B.); 2 Group of Neurodegenerative Diseases, Hospital Universitario 12 de Octubre Research Institute (imas12), 28041 Madrid, Spain; fbartolome.imas12@h12o.es (F.B.); carolinaalquezar.imas12@h12o.es (C.A.); 3 Department of Clinical Research, NeuroTrax Corporation, Modiin 7171102, Israel; glen.doniger@neurotrax.com; 4 Department of Internal Medicine, 12 de Octubre University Hospital, 28041 Madrid, Spain; mariaantonia.nogales@salud.madrid.org (M.A.N.); montserrat.morales@salud.madrid.org (M.M.); 5 Department of Psychiatry, 12 de Octubre University Hospital, 28041 Madrid, Spain; sasesmero@gmail.com; 6 Network Center for Biomedical Research in Neurodegenerative Diseases (CIBERNED), 28029 Madrid, Spain; 7 Division of Neurology, Department of Medicine, Queen’s University, Kingston, ON K7L 2V7, Canada; dlahiri1988@gmail.com; 8 ETSIDI-Center for Automation and Robotics, Universidad Politécnica de Madrid, 28012 Madrid, Spain; cecilia.garcia@upm.es; 9 Department of Medicine, Faculty of Medicine, Complutense University, 28040 Madrid, Spain

**Keywords:** toxic oil syndrome, cognitive impairment, case-control study, long-term effects, fatigue, depression, anxiety, neurotoxicity

## Abstract

**Background:** Toxic oil syndrome (TOS) was a major food-borne epidemic that occurred in Spain in May 1981, caused by the ingestion of rapeseed oil adulterated with aniline. While the somatic sequelae of TOS have been well documented, its long-term cognitive consequences remain poorly understood more than four decades after exposure. **Methods:** In this case-control study, 50 individuals with clinically confirmed TOS were compared to 50 healthy controls matched for age, sex, and education. All participants completed a comprehensive neuropsychological assessment, along with questionnaires evaluating fatigue, anxiety, depression, and health-related quality of life. Multivariate regression models were adjusted for demographic and vascular risk factors, as well as for mood symptoms, fatigue, and use of central nervous system-acting medications. Structural equation modeling was used to explore the potential mediating effects of affective and fatigue symptoms on cognitive performance. **Results:** TOS survivors showed significantly poorer performance than controls in attention, executive function, processing speed, and global cognition after adjusting for demographic and vascular risk factors. However, these differences were no longer statistically significant after additional adjustment for fatigue, depression, anxiety, and central nervous system-acting medications. Structural equation modeling analyses revealed that affective symptoms—particularly fatigue—substantially mediated the relationship between TOS and cognitive performance. **Conclusions:** The cognitive profile observed mirrors that of disorders characterized by subcortical dysfunction and impaired neural connectivity, such as multiple sclerosis and vascular cognitive impairment. Although early postmortem studies in TOS did not demonstrate overt white matter lesions, our findings raise the possibility of long-lasting alterations involving both white and gray matter networks. These results emphasize the need to consider mood and fatigue symptoms when evaluating cognition in TOS survivors and point to the potential for widespread, enduring neurobiological effects stemming from the original toxic exposure.

## 1. Introduction

Toxic oil syndrome (TOS) emerged as one of the most devastating public health crises in contemporary Spanish history, first identified in May 1981 during a massive outbreak of food poisoning. The source was traced to rapeseed oil denatured with aniline—intended for industrial use but fraudulently marketed as olive oil for human consumption [1,2]. The epidemic affected over 20,000 individuals and resulted in more than 300 deaths within the first year alone [2,3]. Survivors experienced a wide range of chronic, multisystemic sequelae, the most prominent being muscle atrophy, typically secondary to an eosinophilic inflammatory myopathy [3,4,5,6]. Other long-term complications included myalgias, muscle cramps [7], severe weight loss, pulmonary hypertension, scleroderma-like syndromes, joint contractures, Sjögren’s syndrome, alopecia, pruritus, and chronic hepatitis [3].

Neurological involvement has been consistently observed in this population and can be divided into two major domains. The first involves the peripheral nervous system, with symptoms such as numbness, paresthesia, and hypoesthesia attributed to inflammatory neuropathy and perineural fibrosis [4,5,7]. The second includes central nervous system manifestations—insomnia, chronic headache, and memory disturbances—suggesting a potential central neurotoxic process [7,8].

Cognitive impairment is one of the most functionally significant yet understudied sequelae among TOS survivors and was already evident in the early stages of the disease [8]. Deficits have been reported in both short- and long-term memory—particularly affecting semantic and episodic domains across verbal and non-verbal formats [8]. Patients exhibited reduced attention, poor concentration, mental fatigue, and psychomotor slowing, with notably slower processing speed and delayed reaction times [7,8]. 

Long-term follow-up studies have shown that many TOS survivors continue to experience enduring neuromuscular and psychological symptoms decades after the acute phase. In cohorts followed for 8 to 12 years, a substantial proportion of patients reported persistent fatigue, muscle cramps, arthralgias, Raynaud’s phenomenon, subjective cognitive complaints, and psychiatric symptoms [6,9]. While some early complications—such as pulmonary hypertension and scleroderma-like skin changes—tended to stabilize or regress over time, chronic morbidity remained common [6,9]. Additionally, metabolic abnormalities, including hypercholesterolemia and hyperglycemia, were frequently observed [9]. These findings support the characterization of TOS as a chronic, multisystemic disorder with a fluctuating course and incomplete resolution in many individuals [6,9,10].

Despite the magnitude of the epidemic and the chronicity of its effects, the long-term cognitive trajectory of TOS survivors has been poorly characterized. Indeed, no studies have evaluated cognitive function in this population more than four decades after the exposure.

In this context, the present study aims to assess whether individuals affected by TOS continue to exhibit measurable cognitive deficits 43 years after the outbreak. While earlier studies have identified neurocognitive alterations in this population [7,8], it remains unclear whether these impairments persist, progress, or resolve over time when compared to demographically matched individuals from the general population.

## 2. Methods

### 2.1. Standard Protocol Approvals, Registrations, and Patient Consents

The ethical standards committees approved all procedures on human experimentation at the 12 de Octubre University Hospital, Madrid, Spain (CEIC codes: 17/035 and 23/616). We obtained written (signed) informed consent from all participants.

### 2.2. Study Design and Setting

Between April and June 2024, all TOS case participants and healthy controls were recruited from the province of Madrid, one of the regions most severely affected during the 1981 epidemic.

We designed the study as a case-control study, with the exposed group consisting of individuals diagnosed with TOS and the unexposed group comprising healthy controls. All interviews, cognitive assessments, and study procedures were conducted at the 12 de Octubre University Hospital in Madrid, Spain.

### 2.3. Participants

TOS patients were defined using the same diagnostic criteria applied in previous studies [11]. Eligible participants included individuals who had experienced either the acute or chronic phase of the disease. The acute phase was characterized by alveolar-interstitial pulmonary infiltrates and/or pleural effusion in the presence of absolute eosinophilia (>500 cells/mm^3^). The chronic phase was defined by the presence of myalgia and eosinophilia and/or one or more of the following clinical features clearly attributable to TOS: scleroderma-like skin changes, peripheral neuropathy, pulmonary hypertension, or hepatopathy.

We recruited TOS patients from the monographic clinical unit dedicated to this syndrome at the 12 de Octubre University Hospital in Madrid—currently, the only specialized unit in Spain exclusively devoted to the long-term management of these patients.

Patients were contacted consecutively until the target sample size of 50 participants was achieved. The referent (unexposed) group was recruited from friends and acquaintances residing in the same geographic area. The exposed group included 50 adults who had been exposed to toxic oil 43 years earlier and had developed clinically confirmed TOS. These individuals were frequency-matched to 50 unexposed referents by age (±5 years), sex, and educational level.

A post hoc power analysis indicated that the sample size (*n* = 50 per group) achieved 87.85% power to detect a standardized mean difference of 0.4 (two-tailed *α* = 0.05), observed in the Global Cognitive Score, the study’s primary cognitive outcome.

Patients were excluded if they had a diagnosis of neurodegenerative disorders (e.g., Alzheimer’s disease, Parkinson’s disease), renal disease, cerebrovascular accidents, chronic alcoholism, or any traumatic injury involving the brain, spinal cord, or peripheral nervous system. Referent (control) participants were required to meet the same exclusion criteria as the exposed group.

### 2.4. Measurements

#### 2.4.1. Demographic and Clinical Data

Participant data—including age, sex, educational level, medical history, and current pharmacological treatments—were collected using a standardized clinical questionnaire. Educational level was initially recorded in four categories: “incomplete primary”, “primary”, “secondary or higher”, and “university”. For analytical purposes, these were subsequently dichotomized as “primary or below” (including those with completed primary and those with no formal or incomplete primary schooling) versus “secondary or higher education”. Particular attention was paid to the documentation of medications with potential cognitive effects, specifically central nervous system (CNS)-acting drugs, including anxiolytics, stimulants, antipsychotics, antidepressants, antihistamines, and antiepileptic medications.

#### 2.4.2. Fatigue Measurement

Fatigue was assessed using the Fatigue Impact Scale for Daily Use (D-FIS), a brief eight-item self-report questionnaire specifically designed to evaluate the perceived impact of fatigue on daily functioning [12,13]. Each item is rated on a 5-point scale ranging from 0 (“no problem”) to 4 (“extreme problem”), with higher total scores indicating greater fatigue-related interference [12,13].

#### 2.4.3. Health-Related Quality of Life Assessment

Health-related quality of life was measured using the EuroQol instrument, a well-established and validated generic tool designed to assess perceived health status in healthy individuals and patients with a wide range of medical conditions [14]. The EuroQol consists of two components [14]. The first, known as the EQ-5D descriptive system, includes five items that assess current health problems across five dimensions: mobility, self-care, usual activities, pain/discomfort, and anxiety/depression. Each dimension is rated using three ordinal response levels: (1) no problems, (2) moderate problems, and (3) severe problems. These responses yield a health profile that can describe up to 243 unique health states. EQ-5D index values are calculated according to standardized European algorithms [14], producing a single utility score where 1 indicates full health, 0 represents death, and negative values (with a minimum of −0.109) reflect health states perceived as worse than death. The second component of the EuroQol is the EQ visual analog scale (EQ VAS), a vertical scale ranging from 0 (worst imaginable health state) to 100 (best imaginable health state), on which respondents provide a subjective rating of their overall health status.

#### 2.4.4. Depressive Symptoms

Depressive symptoms were assessed using the Beck Depression Inventory-II (BDI-II) [15], a validated self-report instrument designed to measure the severity of depression in adults. It consists of 21 items, each reflecting a symptom or attitude associated with depression (e.g., sadness, pessimism, fatigue, changes in sleep or appetite). Respondents rate each item on a 4-point scale ranging from 0 (no symptom) to 3 (severe symptom) based on their experiences over the previous two weeks. Total scores range from 0 to 63, with higher scores indicating greater symptom severity [15].

#### 2.4.5. Anxiety Symptoms

Anxiety symptoms were assessed using the Beck Anxiety Inventory (BAI) [16], a widely used self-report questionnaire designed to evaluate the severity of common anxiety symptoms. Respondents rate how much they have been bothered by each symptom over the past week on a 4-point scale, from 0 (“not at all”) to 3 (“severely—I could barely stand it”). The total score ranges from 0 to 63, with higher scores indicating more severe anxiety [16].

#### 2.4.6. Cognitive Performance

Cognitive functioning was assessed using NeuroTrax digital testing [17]. This platform enables standardized, comprehensive cognitive testing in clinical settings and has demonstrated validity and reliability across a range of populations [17,18,19,20,21,22,23]. Due to time constraints, testing was limited to specific cognitive domains: memory (verbal and non-verbal), attention (Go-NoGo and Stroop Interference tasks), information processing speed, executive function (Go-NoGo, Stroop Interference, and Catch Game). All test instructions were presented in Spanish, the participants’ primary language.

### 2.5. Statistical Analyses

Statistical analyses and figure generation were performed using Python (version 3.12.2) and R (version 4.4.2). The following Python packages were used: *pandas* (v2.2.3) for data handling, *TableOne* (v0.9.1) for descriptive statistics, *statsmodels* (v0.14.4) for regression modeling, and *semopy* (v2.3.11) for structural equation modeling.

In this study, cognitive scores were normalized using the mean and standard deviation of the control group. The same approach was applied to depression, anxiety, and fatigue scales. Z-scores were calculated for each participant in both groups. As in prior studies using NeuroTrax, normalized scores from individual test measures (e.g., accuracy, response time) were averaged to generate domain-specific index scores, which were then averaged to produce a global cognitive score [17,18,19].

First, a descriptive analysis of the study population was conducted. Group homogeneity was assessed using appropriate parametric or non-parametric tests based on variable distribution. Subsequently, univariate analyses were performed to compare the two groups on the BDI-II, BAI, D-FIS, and EQ-5D index values, as well as on each NeuroTrax cognitive domain index and global cognitive score.

A series of multivariate models were constructed to assess the impact of different variables on cognitive performance. Initially, well-established confounders from previous literature were included [24,25,26,27], followed by the incorporation of intermediate clinical variables such as hypertension and diabetes. In a final model, affective variables (BDI-II and BAI) and fatigue (D-FIS) were added. A structural equation modeling approach was applied to estimate both the direct and indirect effects of these variables on cognitive outcomes.

## 3. Results

Data from 50 patients with TOS) and 50 matched healthy controls were analyzed (Table 1). No statistically significant differences were observed between the two groups in terms of sex distribution, age, or educational level. However, TOS patients had a significantly higher prevalence of arterial hypertension (66% vs. 18%; *p* < 0.001) and diabetes mellitus (24% vs. 6%; *p* = 0.025), as well as greater use of CNS-acting medications (58% vs. 20%; *p* < 0.001).

**Figure 1:**
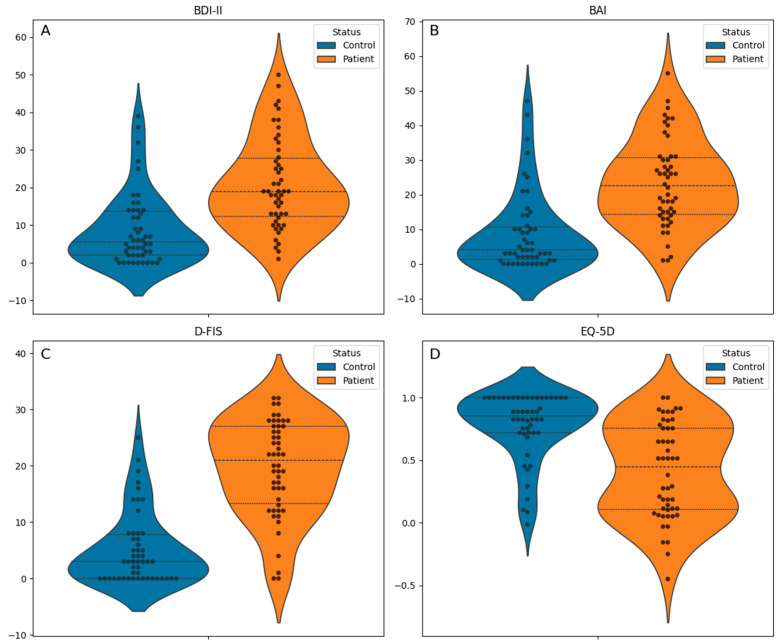
Group differences between patients and controls on the Beck Depression Inventory-II (BDI-II), Beck Anxiety Inventory (BAI), Fatigue Impact Scale for Daily Use (D-FIS), and EQ-5D. Data are visualized using swarm plots overlaid with medians and interquartile ranges (Q1–Q3). Higher scores on BDI-II, BAI, and D-FIS indicate greater symptom severity, while lower EQ-5D index values reflect poorer health-related quality of life.

Regarding affective and fatigue symptoms (Figure 1), TOS patients reported significantly more symptoms across all scales: BDI-II (median 19.0 [interquartile range (IQR) 12.2–27.8] vs. 5.5 [IQR 2.0–13.8]; *p* < 0.001; Figure 1A), BAI (median 22.5 [IQR 14.2–30.8] vs. 4.0 [IQR 1.2–10.8]; *p* < 0.001; Figure 1B), and D-FIS (mean 19.7 ± 8.5 vs. 5.2 ± 6.3; *p* < 0.001; Figure 1C). Their health-related quality of life, assessed using the EQ-5D index, was also significantly lower (median 0.4 [IQR 0.1–0.8] vs. 0.9 [IQR 0.7–1.0]; *p* < 0.001; Figure 1D). A summary of demographic and clinical characteristics is provided in Table 1.

**Figure 2:**
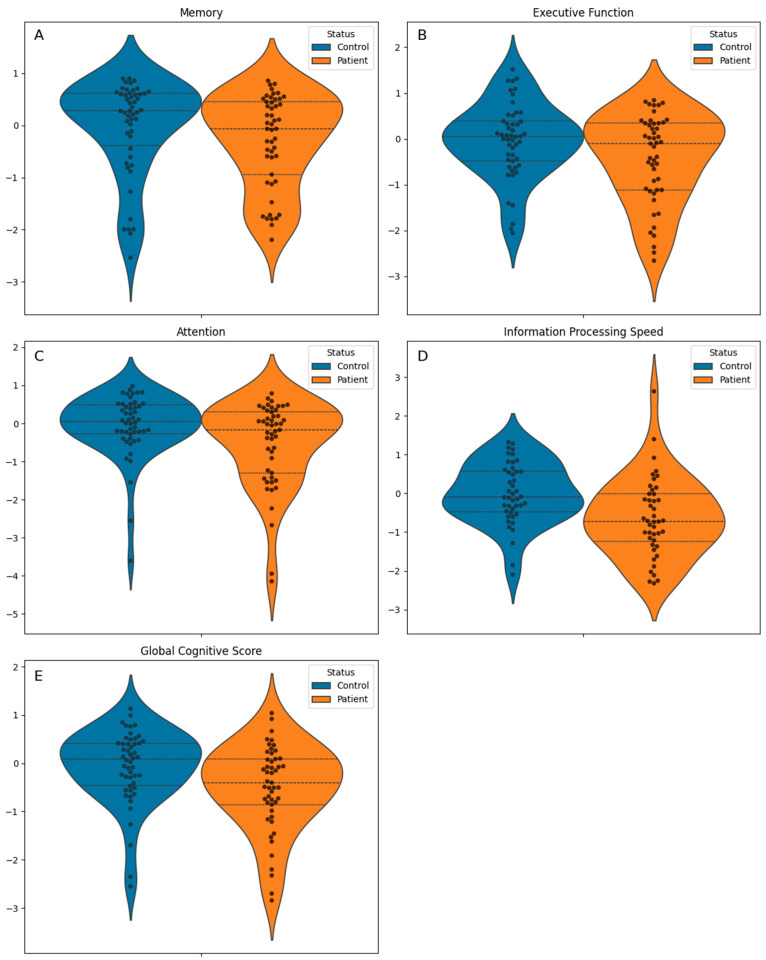
Comparison of cognitive performance between groups (toxic oil syndrome patients and controls). Individual test scores were transformed into z-scores using the mean and standard deviation of the control group as the reference. Domain index scores were calculated by averaging the contributing standardized test scores, and the global cognitive score was calculated as the average of all domain index scores (see Section 2). Boxplots display the median and interquartile range (IQR; Q1 to Q3), with overlaid swarm plots to illustrate individual data points. Higher z-scores reflect better cognitive performance.

Among the cognitive scores, no statistically significant differences were found for memory (median z-score: −0.1 [IQR −0.9 to 0.4] vs. 0.3 [IQR −0.4 to 0.6]; *p* = 0.07; Figure 2A) irrespective of whether immediate and delayed portions were combined or analyzed separately. However, TOS patients exhibited significantly poorer performance in executive function (mean z-score: −0.4 ± 1.0 vs. 0.0 ± 0.8; *p* = 0.036; Figure 2B), attention (median z-score: −0.2 [IQR −1.3 to 0.3] vs. 0.1 [IQR −0.3 to 0.5]; *p* = 0.024; Figure 2C), information processing speed (mean z-score: −0.6 ± 1.0 vs. 0.0 ± 0.8; *p* = 0.002; Figure 2D), and on the global cognitive score (mean z-score: −0.48 ± 0.18 vs. 0.05 ± 0.20; *p* < 0.001; Figure 2E), compared to controls. The distribution of these cognitive outcomes is illustrated in Figure 2 and Table 1.

The models were adjusted for demographic variables (age, age squared, sex, educational level) and relevant medical history (arterial hypertension, diabetes mellitus), consistent with previous evidence linking these factors to cognitive outcomes [28,29,30]. The inclusion of arterial hypertension and diabetes mellitus was further justified by their higher prevalence in our sample, as well as in the broader population affected by TOS [31].

The multivariate models (Table 2) revealed significant associations between TOS diagnosis and reduced cognitive performance. Specifically, TOS was associated with poorer memory (β = −0.307, *p* = 0.050) and information processing speed (β = −0.606, *p* = 0.002) as well as lower global cognitive scores (β = −0.382, *p* = 0.006).

In a subsequent step, additional variables—including fatigue (D-FIS), depressive symptoms (BDI-II), anxiety (BAI), and the use of CNS-acting medications—were incorporated to assess potential confounding effects.

Multicollinearity among the independent variables was evaluated using the variance inflation factor. No evidence of severe multicollinearity was found among the affective variables; however, a variance inflation factor of 6.77 between BAI and BDI-II suggested moderate collinearity. Pearson correlation analyses revealed a strong positive correlation between fatigue and BAI (*r* = 0.72) and between fatigue and BDI-II (*r* = 0.72). Additionally, Spearman correlation coefficients between CNS-acting medication use and the affective scales indicated moderate associations: ρ = 0.36 with fatigue, ρ = 0.38 with depression, and ρ = 0.32 with anxiety.

**Figure 3:**
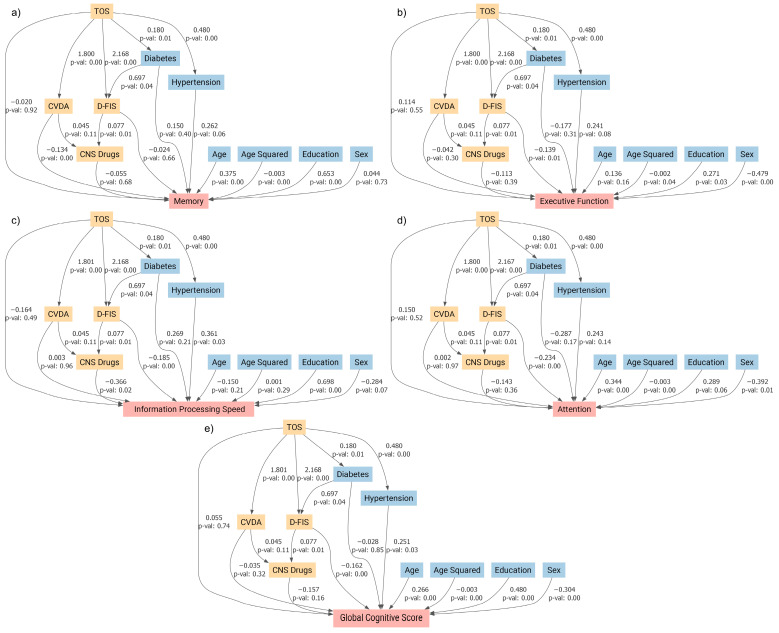
Structural equation modeling diagrams illustrating the relationships between toxic oil syndrome (TOS) and performance on five cognitive outcomes: (**a**) memory, (**b**) attention, (**c**) executive function, (**d**) information processing speed, and (**e**) global cognitive score. The models include the following predictors: TOS diagnosis, sex (female), use of central nervous system-acting medications (CNS Drugs), composite variable for depression and anxiety (CVDA), and Fatigue Impact Scale for Daily Use (D-FIS). Direct and indirect effects are shown, with uncorrected *p*-values reported for each path.

To minimize collinearity issues, we derived a composite variable from the BAI and BDI-II scores using principal component analysis. This principal component accounted for 90.9% of the shared variance between the two scales, capturing the underlying affective dimension. This composite variable, together with the D-FIS, was incorporated into separate structural equation models for each cognitive domain score. The resulting models are depicted in Figure 3.

To explore the relationships among variables and construct the structural equation models, multiple linear regression analyses were performed using the composite depression/anxiety variable, D-FIS, and the use of CNS-acting medications as independent variables. The results of these models are shown in Table 3.

In these models (Figure 3), the previously observed significant impact of a TOS diagnosis on cognitive performance disappeared. However, affective variables emerged as significant predictors across various cognitive domains. Fatigue showed a significant association with attention, executive function, information processing speed, and the global cognitive score (all *p* < 0.01), whereas the composite depression/anxiety variable was significantly associated with memory performance (*p* < 0.01). Notably, the use of CNS-acting medications did not have a direct significant effect on cognitive function in any of the models.

In the mediation analysis of these models (Table 4), mediated proportions near or exceeding 1 were observed, suggesting that the effects of these confounding variables largely account for the association between TOS and cognitive performance.

The mediation effect of affective variables on information processing speed was comparatively lower (72.9%) than that of other cognitive subscales, with fatigue accounting for most of the effect (66.3% of the total). Notably, the direct influence of TOS was not statistically significant in this model.

## 4. Discussion

After adjusting for demographic and clinical variables, TOS survivors demonstrated significantly poorer cognitive performance than matched controls. Consistent with prior findings [9,31], they also exhibited elevated levels of depression, anxiety, and fatigue. However, once affective symptoms, fatigue, and CNS-acting medications were included in the models, the cognitive differences between groups became non-significant, indicating that these variables account for much of the observed impairment. Structural equation modeling confirmed that fatigue was the most influential mediator linking TOS exposure to cognitive outcomes.

These findings suggest that the long-term cognitive profile of TOS survivors may be shaped more by persistent psychological and functional sequelae than by overt structural brain damage. Nonetheless, such symptoms likely reflect downstream effects of chronic neurotoxic exposure, possibly mediated by enduring disruptions in brain network connectivity.

The cognitive deficits identified—primarily in processing speed, executive function, and attention—mirror those seen in subcortical disorders such as multiple sclerosis [32,33], CADASIL [34,35], vascular cognitive impairment [36], and mild traumatic brain injury [37,38,39], pointing toward shared neurobiological underpinnings. Both fatigue and depression are frequent in these conditions and are well-documented contributors to cognitive impairment [40,41], with depression further recognized as a risk factor for Alzheimer’s disease and vascular dementia [42,43].

In this context, fatigue played a particularly central role in our cohort, with robust effects on attention, executive function, information processing speed, and the global cognitive score. These findings are in line with evidence from post-COVID syndrome, where mental fatigue is closely linked to reduced alertness and cognitive slowing [44]. Conversely, depression and anxiety more commonly correlate with memory dysfunction, as documented in psychiatric populations [45] and in post-COVID cohorts with elevated affective burden [46]. Supporting this, previous studies have demonstrated that fatigue contributes to deficits in verbal memory and planning [47] and is associated with dysfunction in cortico-striatal circuits that regulate effort, reward processing, and executive control [48]. In CADASIL and other subcortical disorders, fatigue and depression often co-occur and interact bidirectionally, further complicating their cognitive impact [35]. 

Neuropathological evidence in TOS supports a central nervous system component, including central chromatolysis in anterior horn cells and brainstem nuclei [5,49], as well as changes in key structures such as the locus coeruleus, raphe nuclei, and medullary reticular formation, alongside non-necrotizing vasculitis and focal ischemic lesions in severe cases [4,5]. Although early postmortem studies did not identify overt white matter damage [4,5], the broader involvement of CNS regions suggests a mechanism that extends beyond focal lesions and may account for the persistent cognitive dysfunction observed more than four decades post-exposure.

While the loss of group-level cognitive differences after adjusting for affective symptoms might suggest a psychological origin, it is more plausible that these symptoms reflect chronic neurobiological consequences of the original toxic exposure. Long-term alterations in brain network connectivity—similar to those reported in CADASIL [50], vascular dementia [51], and multiple sclerosis [52]—may underlie the persistent cognitive deficits observed in TOS survivors. Moreover, disrupted connectivity has also been extensively documented in affective disorders such as depression [53,54,55,56], further supporting the notion of shared neural substrates across cognitive and emotional domains.

This study has several limitations. First, its cross-sectional design precludes causal inference. Longitudinal studies are required to establish the directionality between affective symptoms, fatigue, and cognitive decline. Second, the absence of neuroimaging data limits our ability to relate cognitive changes to structural or functional brain alterations. Third, multicollinearity among affective and fatigue measures may have biased model estimates—particularly in domains like attention and processing speed, which are highly sensitive to central fatigue. Although we applied principal component analysis and built separate models to address this, residual confounding cannot be excluded. In addition, reliance on self-reported questionnaires introduces the possibility of reporting bias. Fourth, some mediation models yielded proportions exceeding 100%, potentially reflecting suppressor effects, model misspecification, or unmeasured variables. Fifth, control participants were recruited from acquaintances of TOS survivors, which, despite improving socio-demographic matching, may have introduced selection bias or overmatching due to shared exposures. Nonetheless, this approach was pragmatic, given the syndrome’s rarity and latency. Finally, the modest sample size may have limited our power to detect subtle cognitive differences.

In summary, TOS survivors continue to exhibit long-term cognitive deficits, particularly in processing speed, attention, and executive function. These impairments appear to be largely mediated by fatigue and mood symptoms, which likely reflect the neurobiological consequences of the original toxic exposure. Rather than resulting from discrete structural lesions, the cognitive sequelae may be driven by chronic disruption in large-scale brain connectivity. Clinically, these findings highlight the importance of routinely assessing and treating fatigue and affective symptoms in TOS survivors, as these modifiable factors may significantly impact cognition and quality of life. Future studies incorporating neuroimaging, longitudinal tracking, and digital cognitive tools such as eye-tracking technologies will be critical for disentangling the complex pathophysiology underlying these outcomes.

## Figures and Tables

**Table 1:** Demographic, clinical, and neuropsychological characteristics of study participants.

Variable	Overall (*n* = 100)	Control (*n* = 50)	Patient (*n* = 50)	*p* Value
Sex, *n* (%)				0.284 ^a^
Male	32 (32.0)	19 (38.0)	13 (26.0)	
Female	68 (68.0)	31 (62.0)	37 (74.0)	
Age, mean (standard deviation)	59.3 (8.0)	58.7 (8.2)	59.9 (7.8)	0.449 ^b^
Education, *n* (%)				0.546 ^a^
Illiterate or primary studies	44 (44.0)	20 (40.0)	24 (48.0)	
Secondary or higher	56 (56.0)	30 (60.0)	26 (52.0)	
Central nervous system-acting medications, *n* (%)	39 (39.0)	10 (20.0)	29 (58.0)	<0.001 ^a^
Arterial hypertension, *n* (%)	42 (42.0)	9 (18.0)	33 (66.0)	<0.001 ^a^
Diabetes mellitus, *n* (%)	15 (15.0)	3 (6.0)	12 (24.0)	0.025 ^a^
EQ-5D index, median [Q1, Q3]	0.7 [0.3, 0.9]	0.9 [0.7, 1.0]	0.4 [0.1, 0.8]	<0.001 ^c^
Fatigue Impact Scale for Daily Use, mean (standard deviation)	12.4 (10.4)	5.2 (6.3)	19.7 (8.5)	<0.001 ^b^
Beck Anxiety Inventory, median [Q1, Q3]	14.0 [3.0, 26.0]	4.0 [1.2, 10.8]	22.5 [14.2, 30.8]	<0.001 ^c^
Beck Depression Inventory, median [Q1, Q3]	13.0 [4.8, 21.0]	5.5 [2.0, 13.8]	19.0 [12.2, 27.8]	<0.001 ^c^
Global Cognitive Score, mean (standard deviation)	−0.3 (0.9)	−0.1 (0.8)	−0.5 (0.9)	0.010 ^b^
Cognitive domains				
Memory, median [Q1, Q3]	0.2 [−0.6, 0.5]	0.3 [−0.4, 0.6]	−0.1 [−0.9, 0.4]	0.070 ^c^
Executive function, mean (standard deviation)	−0.2 (0.9)	−0.0 (0.8)	−0.4 (1.0)	0.036 ^b^
Attention, median [Q1, Q3]	−0.0 [−0.5, 0.4]	0.1 [−0.3, 0.5]	−0.2 [−1.3, 0.3]	0.024 ^c^
Information processing speed, mean (standard deviation)	−0.3 (1.0)	−0.0 (0.8)	−0.6 (1.0)	0.002 ^b^

^a^ Chi-square test; ^b^ Student t test; ^c^ Mann-Whitney U test.

**Table 2:** Multivariable linear regression models examining the association of toxic oil syndrome diagnosis with cognitive performance, adjusted for demographic and clinical covariates.

Predictor Variable	Global Cognitive Score Coefficient (*p* Value)	Memory Coefficient (*p* Value)	Executive Function Coefficient (*p* Value)	Attention Coefficient (*p* Value)	Information Processing Speed Coefficient (*p* Value)
Diabetes mellitus	–0.183 (0.294)	0.044 (0.824)	–0.318 (0.107)	–0.472 (0.050)	0.170 (0.523)
Educational level	0.437 (0.001)	0.657 (<0.001)	0.229 (0.119)	0.213 (0.231)	0.644 (0.001)
Age squared	–0.002 (0.019)	–0.003 (0.004)	–0.001 (0.271)	–0.003 (0.021)	–0.000 (0.910)
Age	0.176 (0.071)	0.284 (0.012)	0.057 (0.603)	0.254 (0.058)	–0.020 (0.904)
Arterial hypertension	0.220 (0.115)	0.262 (0.101)	0.218 (0.164)	0.199 (0.295)	0.215 (0.273)
Sex (female)	–0.356 (0.006)	0.025 (0.864)	–0.517 (<0.001)	–0.453 (0.010)	–0.439 (0.018)
Toxic oil syndrome diagnosis	–0.382 (0.006)	–0.307 (0.050)	–0.274 (0.074)	–0.369 (0.048)	–0.606 (0.002)

Coefficients and *p*-values from multivariable linear regression models assessing the association of demographic and clinical variables (diabetes mellitus, educational level, age squared, age, arterial hypertension, sex, toxic oil syndrome diagnosis) with five cognitive measures: global cognition, memory, executive function, attention, and information processing speed. All models were adjusted for the full set of listed covariates.

**Table 3:** Associations of clinical and demographic variables with affective symptoms, fatigue, and CNS-acting medication use.

Predictor Variable	Depression/Anxiety Composite Coefficient	*p* Value	Fatigue Impact Scale for Daily Use Coefficient	*p* Value	Use of Central Nervous System-Acting Medications Coefficient	*p* Value
Diabetes mellitus	0.590	0.187	4.561	0.042	0.102	0.457
Educational level	0.020	0.951	2.620	0.108	–0.067	0.502
Age	–0.003	0.900	0.020	0.843	0.002	0.742
Arterial hypertension	–0.003	0.994	1.345	0.445	0.024	0.824
Sex (female)	0.040	0.902	1.304	0.418	0.117	0.243
Toxic oil syndrome diagnosis	1.695	<0.001	13.062	<0.001	0.328	0.003

Coefficients and *p*-values from linear regression models evaluating the associations of clinical and demographic predictors (diabetes mellitus, educational level, age, arterial hypertension, sex, and toxic oil syndrome diagnosis) with three outcomes: composite depression/anxiety score, Fatigue Impact Scale for Daily Use, and central nervous system-acting medications.

**Table 4:** Mediation effects of affective variables, fatigue, and CNS-acting medications on the relationship between TOS and cognitive performance.

	Global Cognitive Score	Memory	Attention	Executive Function	Information Processing Speed
Average Direct Effect	0.055	−0.02	0.15	0.114	−0.164
Average Causal Mediation Effects (composite variable of depression/anxiety)	−0.062	−0.241	0.003	−0.076	0.005
Average Causal Mediation Effects (Fatigue Impact Scale for Daily Use)	−0.351	−0.051	−0.508	−0.302	−0.402
Average Causal Mediation Effects (Central Nervous System-Acting Medications)	−0.019	−0.007	−0.018	−0.014	−0.045
Total Effect	−0.377	−0.318	−0.372	−0.278	−0.606
Mediated proportion	1.146	0.938	1.404	1.409	0.729

Average direct effect, average causal mediation effects, total effect, and mediated proportion of the association between toxic oil syndrome and cognitive performance, considering mediation through three variables: the composite depression/anxiety score, fatigue (Fatigue Impact Scale for Daily Use), and central nervous system-acting medications. Cognitive outcomes include global cognitive score, memory, attention, executive function, and information processing speed. Negative values indicate poorer performance.

## Data Availability

The original contributions presented in this study are included in the article. Further inquiries can be directed to the corresponding author.
